# The *in-vitro *evaluation of antibacterial, antifungal and cytotoxic properties of *Marrubium vulgare *L. essential oil grown in Tunisia

**DOI:** 10.1186/1476-511X-10-161

**Published:** 2011-09-21

**Authors:** Zied Zarai, Adel Kadri, Ines Ben Chobba, Riadh Ben Mansour, Ahmed Bekir, Hafedh Mejdoub, Néji Gharsallah

**Affiliations:** 1 Laboratoire de Biochimie et de Génie Enzymatique des Lipases, ENIS, BPW, 1173 Sfax, University of Sfax, Tunisia; 2 Laboratoire de Chimie des Substances Naturelles, Faculté des Sciences de Sfax, B.P. 1171, 3000 Sfax, University of Sfax, Tunisia; 3 Laboratoire de Microbiologie Alimentaire, Faculté des sciences de Sfax, B.P. 1171, 3000 Sfax, University of Sfax Tunisia; 4 Unité de recherche Biotechnologie et pathologies, Institut Supérieur de Biotechnologie de Sfax, University of Sfax, Tunisia; 5 Département de Génie des procédés, ISET Sfax, Km 2,5 Rte de Mahdia, 3099 Sfax, University of Sfax, Tunisia; 6 Laboratoire de Biochimie, Faculté des sciences de Sfax, B.P. 1171, 3000 Sfax, University of Sfax, Tunisia

**Keywords:** Antimicrobial, cytotoxicity, essential oil, *Marrubium vulgare *L.,, pathogenic microorganisms, HeLa cell lines

## Abstract

**Background:**

In order to validate its antiseptic and anticancer properties with respect to traditional uses, we have screened for the first time the antimicrobial activity of aerial parts of *M. vulgare *L. essential oil against different pathogenic microorganisms and the cytotoxic activity against HeLa cell lines.

**Methods:**

The agar disk diffusion method was used to study the antibacterial activity of *M. vulgare *essential oil against 12 bacterial and 4 fungi strains. The disc diameters of zone of inhibition (DD), the minimum inhibitory concentrations (MIC) and the concentration inhibiting 50% (IC_50_) were investigated to characterize the antimicrobial activities of this essential oil. The *in vitro *cytotoxicity of *M. vulgare *essential oil was examined using a modified MTT assay; the viability and the IC_50 _were used to evaluate this test.

**Results:**

The antimicrobial activity of the essential oil was investigated in order to evaluate its efficacy against the different tested microorganisms. The present results results showed a significant activity against microorganisms especially Gram (+) bacteria with inhibition zones and minimal inhibitory concentration values in the range of 6.6-25.2 mm and 1120-2600 μg/ml, respectively, whereas Gram (-) bacteria exhibited a higher resistance. As far as the antifungal activity, among four strains tested, *Botrytis cinerea *exhibited the strongest activity with inhibition zones of 12.6 mm. However, *Fusarium solani, Penicillium digitatum *and *Aspergillus niger *were less sensitive to *M. vulgare *essential oil. About the citotoxicity assay, this finding indicate the capability of this essential oil to inhibited the proliferation of HeLa cell lines under some conditions with IC_50 _value of 0.258 μg/ml.

**Conclusion:**

This investigation showed that the *M. vulgare *essential oil has a potent antimicrobial activity against some Gram (+) pathogenic bacteria and *Botrytis cinerea *fungi. The present studies confirm the use of this essential oil as anticancer agent. Further research is required to evaluate the practical values of therapeutic applications.

## Background

The Lamiaceae plants was considered as one of the large plant families used as a framework to evaluate the occurrence of typical secondary metabolites [1]. The genus *Marrubium *comprises 10 species, which are found wild in many regions of Tunisia. Among them, *Marrubium vulgare *L. is a perennial herb of the Labiatae family which is commonly known as "horehound" in Europe, or "Marrubia" in Tunisia, is naturalized in North and South America, the latter and Western Asia. It possesses tonic, aromatic, stimulant, expectorant, diaphoretic and diuretic properties. It is helpful for bronchial asthma and non-productive cough. It was formerly much esteemed in various uterine, visceral and hepatic affections and in phthisis [2]. In the Mediterranean region, *M. vulgare *is frequently used in folk medicine to cure a variety of diseases. The plant is reported to possess hypoglycemic [3], vasorelaxant [4], antihypertensive [5], analgesic [6,7], anti-inflammatory [8], antioxidant activity [9,10], antiedematogenic activity [11], and many other biological activities. In Tunisian folk medicine, it was used as hypotensive, hypoglycemic and cardiotonic.

Recently, a large number of essential (volatile) oils and their constituents have been investigated for their biological activity, notably antibacterial, antifungal, and antioxidant properties [12-14]. Essential oils and their components are gaining increasing interest as a natural alternative to synthetic drugs [15], particularly against microbial agents because of their relatively safe status wide acceptance by consumers and their exploitation for potential multipurpose functional use. The chemical compositions of *M. vulgare *essential oil from various origins have been the subject of many studies. The literature reveals the occurence of several chemotypes. From Lithuania, (Z)-β-farnesene, β-caryophyllene, (E)-2-hexenal, α-humulene and germacrene D were the main components of *M. vulgare *essential oil [16]. From Czech Republic, the main constituents of *M. vulgare *essential oil were *β*-caryophyllene and germacrene D [17]. From different region of Iran, the main constituants of *M. vulgare *essential tricyclene, *β*-pinene, bisabolol, *β*-elemone and isomenthon-8-thiol [18], β-bisabolene, 8-cadinene and isocaryophyllene [19], and bisabolene, *β*-caryophyllene, germacrene D and *E*-*β*-farnesene [20], caryophyllene oxide, *β*-caryophyllene and germacrene D [21].

The interest in plants with antimicrobial properties has been revived because of current problems associated with the use of antibiotics [22]. Therefore, essential oils and other naturally occurring antimicrobials are attractive to the food industry as well as imparting flavor [23]. More recently, the essential oil of this plant was advocated for their use as an antioxidant agent [10], but to the best of our knowledge, there are no reports on the antimicrobial properties and the cytotoxicity has been published.

Therefore, this paper was conducted to investigate for the first time the antimicrobial properties against clinical and pathogenic microorganisms and the cytotoxicity of *M. vulgare *essential oil grown in Tunisia.

## Methods

### Chemicals, reagents and plant material

Chemicals and reagents were supported by Prolabo (Paris, France) and Pharmacia (Uppsala, Swedeen). Plant materials (aerial parts) of *M. vulgare *L. were grown in the vicinity of the village of Ouled Mnasser, with a latitude of 34.88 (34° 52' 60 N) and a longitude of 9.13 (9° 7' 60 E) in Sidi Bouzid, Tunisia. The aerial parts of wild growing plant have been collected during the period of June-July 2009. The plant materials were confirmed by a senior A. bekir. Voucher specimens were deposited at ISET, Sfax (Département de Génie des procédés) as Bekir 520.

### Distillation of essential oil and GC/MS analysis conditions

The fresh aerial parts of *M. vulgare *(300 g) were hydrodistilled using a Clevenger-type apparatus to recover the essential oils for 4 h. The distilled essential oils were dried over anhydrous sodium sulfate, filtered and stored at +4°C.

The essential oil was analyzed using an Agilent-Technologies 6890 N Network GC system equipped with a flame ionization detector and HP-5MS capillary column (30 m × 0.25 mm, film thickness 0.25 μm; Agilent-Technologies, Little Falls, CA, USA). The injector and detector temperatures were set at 250 and 280°C, respectively. The column temperature was programmed from 35 to 250°C at a rate of 5°C/min, with the lower and upper temperatures being held for 3 and 10 min, respectively. The flow rate of the carrier gas (helium) was 1.0 ml/min. A sample of 1.0 μl was injected, using split mode (split ratio, 1:100). All quantifications were carried out using a built-in data-handling program provided by the manufacturer of the gas chromatograph. The composition was reported as a relative percentage of the total peak area. The identification of the essential oil constituents was based on a comparison of their retention times to *n*-alkanes, compared to published data and spectra of authentic compounds. Compounds were further identified and authenticated using their mass spectra compared to the Wiley version 7.0 library.

### Antimicrobial activity assay

#### Microbial strain

The essential oil of *M. vulgare *was individually tested against a panel of microorganisms (Table 1). The antimicrobial activities of essential oil were determined against sixteen of human-pathogenic microbial strains. The bacteria and fungi used were selected because they have implicated with skin, oral and intestinal tract of man. Twelve species of bacteria and four species of fungi as shown in Table 1 were used in this study.

**Table 1:** Pathogenic bacteria and fungi used for the antimicrobial assay.

**Bacteria**	
*Staphylococcus aureus *1327	*Staphylococcus epidermidis*
*Micrococcus luteus*	*Enterococcus faecalis*
*Enterobacter cloacae*	*Staphylococcus aureus *25923
*Bacillus subtilis*	*Bacillus cereus*
*Pseudomonas aeruginosa 27853*	*Klebsielle pneumoniae *WHO24
*Escherchia coli *25922	
**Fungi**	
*Botrytis cinerea*	*Fusarium solan*
*Penicillium digitatum*	*Aspergillus niger*

#### Agar diffusion method

The agar diffusion method was employed for the determination of antibacterial activities of *M. vulgare *essential oil according to the method described by Berghe and Vlietinck (1991) [24]. The essential oil extracts were dissolved in 100% ethanol to a final concentration of 10 mg/ml and sterilized by filtration trough 0.22 μm Nylon membrane filter. The bacterial strains were cultured in a nutriment broth for 24 hours. Then, 200 μl of each suspension bacteria (10^6 ^CFU estimated by absorbance at 600 nm) was spread on Luria Broth agar. Bores were made by using a sterile borer and were loaded with 10 μl of each sample extract. Ethanol was used as negative control and ampicillin (10 μg/puit) as positive reference standard. All the plates were incubated at 37°C for 24 hours. Antibacterial activity was evaluated by measuring the zone of inhibition in millimetres. All experiments were done in triplicates.

#### Determination of the minimal inhibitory concentration (MIC)

The minimal inhibitory concentration (MIC) values, which represent the lowest essential oil concentration that completely inhibits the growth of microorganisms, were determined by a micro-well dilution method [25]. The inoculum of each bacterium was prepared and the suspensions were adjusted to 10^6 ^CFU/ml. All the extracts were dissolved in 100% ethanol and then dilutions series were prepared in a 96-well plate. Each well of the microplate included 40 μl of the growth medium, 10 μl of inoculums and 50 μl of the diluted sample extract. The ampicillin and ethanol are used as positive and negative controls, respectively. The plates were then covered with the sterile plate and incubated at 37°C for 24 h. After that, 40 μl of 3- (4, 5-dimethyl-thiazol-2-yl)-2,5-diphenyl-tetrazolium bromide (MTT) at a final concentration 0.5 mg/ml freshly prepared in water was added to each well and incubated for 30 min. The change to red colour indicated that the bacteria were biologically active. The MIC was taken to the well, where no change of colour of MTT was observed. The MIC values were done in triplicate.

#### Antibacterial assay disc-diffusion method

An antibacterial activity of the essential oils was screened against eight human pathogenic bacteria. The inhibitory effect on bacterial growth was determined using agar-disc diffusion assay [26,27]. The bacterial cultures were first grown on Muller Hinton agar (MH) plates at 37°C for 18 to 24 h prior to seeding onto the nutrient agar. One or several colonies of the respective bacteria were transferred into API suspension medium (bioMerieux) and adjusted to 0.5 McFarland turbidity standards with a Densimat (bioMerieux) [28,29]. The inocula of the respective bacteria were streaked into MH agar plates using a sterile swab and were then dried at 37°C during 15 min. A sterile filter disc having 6 mm of diameter was placed at the surface of MH agar and 5 μl of the essential oil was dropped onto each Whatman paper disc [30]. The treated Petri dishes were incubated at 37°C for 18 to 24 h. The antibacterial activity was evaluated by measuring the clear zone surrounding the Whatman paper. Standard discs of the antibiotic ampicillin were applied as a positive antibacterial controls.

#### Antibacterial assay dilution method

The minimal inhibitory concentration (MIC) of essential oil was determined using the Mueller Hinton broth (MHB) dilution method [31]. All tests were performed in MHB supplemented with ethanol 5% [32]. Bacterial strains were cultured overnight in MHB at 37°C. Tubes of MHB containing various concentrations of oils were inoculated with 10 μl bacterial inoculums adjusted to 10^6 ^CFU/ml. They were incubated under shaking conditions (100-120 rpm) at 37°C for 24 h [33,34]. Control tubes without tested samples were essayed simultaneously. The essays were performed in triplicate. The MIC was defined as the lowest concentration preventing visible growth [35,36].

#### Antifungal assay disc-diffusion method

The biological activity against yeasts was determined by employing disc agar diffusion method using Sabouraud Dextrose agar [37]. An aliquot (5 μl) of the oil was deposited on sterile paper discs (6 mm diameter) which were subsequently placed in the centre of the inoculated Petri dishes. After an incubation period of the 24 h at 30°C, the inhibitory activity was compared to that of commercial cycloheximide at a concentration of 10 mg/ml.

### Cell lines and culture condition

HeLa cells (cervical cancer line, adherent) were used to investigate the cytotoxicity effect of essential oil. This cell line were grown in RPMI 1640 medium (Gibco) supplemented with 10% (v/v) foetal calf serum (FCS) and 2 mM L-glutamin in tissue culture flasks (Nunc). They were passed twice a week and kept at 37°C in a humidified atmosphere of 95% air and 5% CO_2_.

### MTT test

The proliferation rates of HeLa cells after treatment with essential oils were determined by the colorimetric 3-(4,5-dimethylthiazol-2-yl)-2,5-diphenyl tetrazolium bromide (MTT) assay. The yellow compound MTT is reduced by mitochondrial dehydrogenases to the water-insoluble blue compound formazan, depending on the viability of cells.

HeLa cells (4 ×10^4 ^in each well) were incubated in 96-well plates for 24 hours in the presence or absence of essential oil. Twenty microlitres MTT solution (Sigma) (5 mg mL^-1 ^in PBS) were added to each well. The plate was incubated for 4 h at 37°C in a CO_2_-incubator. One hundred and eighty microlitres of medium was removed from every well without disturbing the cell clusters. A 180 μl methanol/DMSO solution (50:50) was added to each well, and the preparations were mixed thoroughly on a plate shaker with the cell containing formazan crystals. After all of the crystals were dissolved, the A570 values were determined with a microplate reader (ELx 800).

## Results and Discussion

### Antimicrobial assays

The antimicrobial activities of *M. vulgare *essential oil against microorganisms examined in the present study and their potency were qualitatively and quantitatively assessed by the presence or absence of inhibition zones and zone diameter (DD), the medium inhibitory concentration (IC_50_) and the minimal inhibitory concentration (MIC) values. This essential oil displayed varied antibacterial and antifungal activities across the studied pathogens. As can be seen from Table 2, essential oil inhibited the growth of bacterial strains produced a zone diameter of inhibition from 6.6 to 25.2 mm for Gram (+) bacteria, along with IC_50 _and MIC values ranging from 560-1100 μg/ml and 1120-2600 μg/ml, respectively. Whereas, for Gram (-) bacteria, no antimicrobial activities of essential oil tested against all strains (*Pseudomonas aeruginosa 27853, Klebsielle pneumoniae *WHO24, *Escherichia coli *25922 *and Salmonella) *has been revealed. Among Gram (+) bacteria, the strongest activity of *M. vulgare *essential oil was observed against *Staphylococcus epidermidis *(25.2 mm) followed by *Staphylococcus aureus 25923 *(18 mm), *Enterobacter cloacae *(13.8 mm), *Bacillus subtilis *(13.2 mm), *Micrococcus luteus* (12 mm) and *Staphylococcus aureus *1327 (12 mm). However, *Enterococcus faecalis *(9.6 mm) and *Bacillus cereus *(6.6 mm) exhibited moderate to weak activities, respectively.

**Table 2:** Antibacterial and antifungal activity of the essential oil of *M. vulgare *using agar disc diffusion, IC_50 _and minimal inhibition concentration (MIC).

Strains	DD^a^	IC_50_^b^	MIC^c^	DD^d^
**Bacterial strains Gram (+)**				
*Staphylococcus aureus*	12.0 ± 0.5	2500 ± 50	>1100.00	20 ± 0.5
*Staphylococcus epidermidis*	25.2 ± 0.3	2200 ± 50	>590.00	26 ± 0.5
*Micrococcus luteus*	12.0 ± 0.5	1120 ± 50	>560.00	20 ± 1.5
*Enterococcus faecalis*	9.6 ± 1.0	1140 ± 10	>680.00	25 ± 1.0
*Enterobacter cloacae*	13.8 ± 0.6	1500 ± 90	>670.00	21 ± 1.4
*Staphylococcus aureus *25923	18 ± 1.0	2600 ± 80	>1150.00	24 ± 0.5
*Bacillus subtilis*	13.2 ± 1.0	2130 ± 50	>890.00	26 ± 0.6
*Bacillus cereus*	6.6 ± 0.4	2113 ± 40	>950.00	21 ± 1.0
**Bacterial strains Gram (-)**				
*Botrytis cinerea*	NS	NS	NS	21 ± 0.5
*Pseudomonas aeruginosa 27853*	NS	NS	NS	20 ± 1.0
*Klebsielle pneumoniae *WHO24	NS	NS	NS	21 ± 0.9
*Escherchia coli *25922	NS	NS	NS	22 ± 0.8
*Salmonella*	NS	NS	NS	29 ± 1.0
**Fungal strains**				
*Botrytis cinerea*	12.6 ± 0.5	2190 ± 12	>1100.00	29 ± 1.0
*Fusarium solani*	6.9 ± 0.5	3220 ± 20	>1190.00	28 ± 0.6
*Penicillium digitatum*	6.6 ± 0.4	3200 ± 15	>1120.00	21 ± 0.9
*Aspergillus. niger*	6.4 ± 0.0	3000 ± 50	>1180.00	30 ± 0.5

Results are means of three different experiments,

^a ^DD: Disc Diameter of inhibition (halo size) in (mm), E. oil 100 μg/disc,

^b ^MIC: minimum inhibitory concentration (μg/ml),

^c^IC_50_: 50% inhibition concentration (μg/ml),

^d^DD: Disc Diameter of inhibition zone of ampicillin (10 μg/disc) and cycloheximide (10 μg/disc), were used as positive controls for bacteria and fungi, respectively,

NS: not sensitive.

For the fungi strains, the disc diameter zones of inhibition ranged from 6.4-12.6 mm, along with IC_50 _and MIC values ranging from 2190-3000 μg/ml and 1100-1180 μg/ml, respectively. The maximal inhibition zones was obtained for *Botrytis cinerea*, however *Fusarium solani*, *Penicillium digitatum *and *Aspergillus niger *exhibited weak activity. Our results suggest that Gram (+) bacteria are more sensitive to the *M. vulgare *essential than Gram (-) bacteria. This was consistent with the previous studies on other spices and herbs [38,39]. This generally higher resistance among Gram (-) bacteria could be ascribed to the presence of their outer membrane, surrounding the cell wall, which restricts diffusion of hydrophobic compounds through its lipopolysaccharide covering. The absence of this barrier in Gram (+) bacteria allows the direct contact of the essential oil's hydrophobic constituents with the phospholipids bilayer of the cell membrane, causing either an increase of ion permeability and leakage of vital intracellular constituents, or impairment of the bacterial enzyme systems. Differences in MIC values of bacteria may be related to the differential susceptibility of bacterial cell wall, which is the functional barrier to minor differences present in the outer membrane in the cell wall composition [40]. The highest sensitivity of *Staphylococcus epidermidis *and *Staphylococcus aureus *may be due to its cell wall structure and outer membrane.

It is most likely that numerous components of the essential oils play a crucial role in defining the features of oils, lipophilic or hydrophilic attraction and fixation on cell wall and membranes, and cellular distribution [41]. This feature is very important because, depending on their component, the distribution of the oil in the cell determines the different types of biological activities such as antibacterial, antifungal and cytotoxicity. As reported previously [10], the *M. vulgare *essential oils isolated by hydrodistillation from the aerial parts was analyzed by HP-5MS column. The general chemical composition of the essential oil, the percentage contents, and retention indices of the components are given in Table 3. Thirty four components could be identified in the oil (100% of the total oil). The essential oil is constituted mainly by approximately equal amounts of oxygenated monoterpenes (40.02%) and sesquiterpenes hydrocarbons (42.70%). The major components were γ-eudesmol (11,93%), followed by β**-**citronellol (9,90%), Citronellyl formate (9,50%) and germacrene D (9,37%). When compared with previous studies [16-21], our study showed that this essential oil possessed an original composition with the main component of γ-eudesmol (11.93%), which is not observed elsewhere.

**Table 3:** Chemical composition, retention indices and percentage composition of the *M. vulgare *essential oil.

N°	Compound	RI	%	Identification
1	N-trimethylsilyl trifluoroacetamide	764	2.35	MS, RI
2	N, N-bis trimethylsilyl trifluoroacetamide	857	0.97	MS, RI
3	α- pinene	932	1.16	MS, RI
4	Camphene	948	0.49	MS, RI
5	1,8-Cineole	1044	3.72	MS, RI
6	α-thujone	1131	2.29	MS, RI
7	1-Vinylcyclohexane	1143	0.75	MS, RI
8	Camphor	1174	1,03	MS, RI
9	Iso menthone	1197	0.57	MS, RI
10	Borneol	1199	0.61	MS, RI
**11**	**β-citronellol**	1266	**9.90**	MS, RI
12	Geraniol	1295	2.74	MS, RI
**13**	**Citronellyl formate**	1315	**9.50**	MS, RI
14	Geranyl formate	1344	6.25	MS, RI
15	α-copaene	1419	1.37	MS, RI
16	β-Bourbonene	1429	1.96	MS, RI
17	trans-caryophyllene	1462	2.15	MS, RI
18	α-Muurolene	1484	0.63	MS, RI
19	α-amorphene	1490	0.81	MS, RI
20	α-Humulene	1495	0.68	MS, RI
21	Neoalloocimene	1502	0.91	MS, RI
22	neryl acetate	1512	3.41	MS, RI
**23**	**Germacrene-D**	1521	**9.37**	MS, RI
24	Ledene	1534	5.35	MS, RI
25	β-bisabolene	1544	0.86	MS, RI
26	δ-cadinene	1559	3.30	MS, RI
27	α -agarofuran	1581	0.42	MS, RI
28	Furan-2-one, 4-phenyltetrahydro	1616	1.44	MS, RI
**29**	**γ-Eudesmol**	1647	**11.93**	MS, RI
30	β-Cubebene	1674	1.52	MS, RI
31	Citronellyl butanoate	1682	0.66	MS, RI
32	Geranyl tiglate	1712	5.53	MS, RI
33	Cyclononasiloxane, octadecamethyl	2198	3.08	MS, RI
34	Eicosamethylcyclodecasiloxane	2264	2.29	MS, RI

Total identification			**100**	
Yield (g/100 g dry weight)			00.34	
Hydrocarbon monoterpenes			01.65	
Oxygenated monoterpenes			40.02	
Hydrocarbon sesquiterpenes			42.70	
Oxygenated sesquiterpene			06.19	

The antimicrobial properties of essential oils from aerial part of *M. vulgare *are suspected to be associated, in part with their high contents of oxygenated compounds (46.21%). Several researchers also report mono- and sesquiterpenoids as the major components of essential oils which are phenolic in nature [42,43]. It is therefore reasonable to assume that their antimicrobial activity might be related to the abundance of phenolic compounds. Referring to the literature, the antimicrobial activity of the tested essential oil can be related to the contribution of the mixture between major (γ-eudesmol, β**-**citronellol, citronellyl formate and germacrene D) and minor (camphene, borneol) [10] constituents, which known to have efficient antimicrobial properties [44,45]. In addition, germacrene-D is known to have a strong effect on insect behavior [46] and has significant antibacterial and antifungal activities [47]. Therefore, Essential oils always represent a complex mixture of different chemical components, thus it is very difficult to reduce the antibacterial effect of the total oil to a few active principles.

Compared to the positive control, ampicillin (belonging to the penicillin group of beta-lactam antibiotics is able to penetrate Gram positive and some Gram negative bacteria) and cycloheximide (generally used only in *in vitro *research applications as a fungicide, and is not suitable for human use as a therapeutic compound) were found to possess lower activity than the tested essential oil. We explain this by the fact, that pure component, such as antibiotics give a more potent antimicrobial activity when compared to a complex mixture of components such as essential oils.

### Cytotoxicity assays

The effect of different concentrations (3.91-3000 μg/ml), of *M. vulgare *essential oil on HeLa cell lines were studied. As depicted in Table 4, they significantly decreased the viability of HeLa in a dose dependent manner. For a concentration up to 250 μg/ml, essential oil destructed HeLa cells by 27%, however for a concentration higher than 500 μg/ml, all HeLa cells were destructed. At lower doses, the oil was tolerated by the cells and its IC_50 _(the concentration of the tested oil required to reduce the cell survival fraction to 50% of the control) was 0.258 μg/ml. The volatile oil displayed an excellent cytotoxic effect towards the human tumor cell line. This makes the tested oil certainly deserve some further investigation. Referring to the literature, the cytotoxicity of the tested essential oils may be due to the presence of some monoterpenes and sesquiterpenes including α-Humulene [48] and isoprenoids including geraniol [49]. These compounds were reported to be active against the tumor cell lines, but in our oil, they exist in small amount [10]. Although, the presence of germacrene D in a good amount can enhances the cytoxicity of the tested oil. This results was confirmed by Setzer et al., who reported that germacrene D has exhibited a 7-fold stronger cytotoxic activity against the Hs 578T cell line in comparison with α-pinene and limonene and a 6-fold stronger action against Hs 578T and Hep-G2 than 1,8-cineole, linalool, 4-terpineol and α-terpineol [50]. Some reports support the relationship of cytotoxicity with antioxidant activity [51]. So the antioxidant activity of *M. vulgare *essential oil might contribute to its cytotoxic activity [45]. Finally, we noted that the synergistic effects of these active chemicals with other constituents of the essential oil should be taken into consideration. This study also justifies and reinforces the use of this plant on traditional medicine. Although all *in vitro *experiments hold limitations with regards to possible *in vivo *efficacy. The results of this study are very promising as it will serve as a data base for researchers in these kinds of studies for indicating which essential oils and plant oils may be useful for specific medical conditions. Furthermore, it is still necessary to investigate in vivo bioactivity and cytoxicity of the oil, to explore in more depth its potential beneficial use in diseases and infections caused by microbes.

**Table 4:** Cytotoxic activity of *M. vulgare *essential oil determined by the MTT assay.

**Oil (**μ**g/ml)**	Mean OD_570 _(nm)	% Viable HeLa cell line
0	0.971	100.00
3.91	0.836	86.10
7.81	0.815	83.93
15.63	0.798	82.18
31.25	0.780	80.94
62.50	0.756	77.85
125	0.717	73.84
250	0.707	72.81
500	0.015	01.54
1000	0.031	00.31
3000	0.023	00.21

## Conclusion

In conclusion, our study can be considered as the first report on the antimicrobial and cytotoxic properties of *M. vulgare *volatile oil. The *in vitro *antimicrobial activity of the obtained may well be due to the presence of synergy, antagonism or additive effects of the tested major components of the oils, which possess various potency of activity. The results of the cytotoxic activity against HeLa cell lines in this study are very promising with regards to possible antineoplastic chemotherapy and form a very sound basis for future research. Our results are a contribution to a better valorization of this medicinal plant. Several other biological tests will be worthwhile to search for more eventual activities of this plant to characterize active principles, and assess toxicity by laboratory assays.

## Competing interests

The authors declare that they have no competing interests.

## Authors' contributions

ZZ, AK, IBC and RBM carried out the experimental part such as extraction, antibacterial, antifungal and cytotoxicity assays. AB contribute to the analysis of the results. HM and NG supervised the work and corrected the manuscript. Authors read and approved the final manuscript.
